# Incorporation of Pentraxin 3 into Hyaluronan Matrices Is Tightly Regulated and Promotes Matrix Cross-linking

**DOI:** 10.1074/jbc.M114.568154

**Published:** 2014-09-04

**Authors:** Natalia S. Baranova, Antonio Inforzato, David C. Briggs, Viranga Tilakaratna, Jan J. Enghild, Dhruv Thakar, Caroline M. Milner, Anthony J. Day, Ralf P. Richter

**Affiliations:** From the ‡ CIC biomaGUNE, 20009 Donostia-San Sebastian, Spain,; the § Humanitas Clinical and Research Center, 20089 Rozzano, Italy,; the ¶ Wellcome Trust Centre for Cell Matrix Research and; the ‡‡ Faculty of Life Sciences, University of Manchester, Manchester M13 9PT, United Kingdom,; the ‖ Department of Molecular Biology and Genetics, University of Aarhus, 8000 Aarhus C, Denmark,; the ** Department of Molecular Chemistry, University Grenoble Alpes and CNRS, 38000 Grenoble, France, and; the §§ Max-Planck-Institute for Intelligent Systems, 70569 Stuttgart, Germany

**Keywords:** Analytical Chemistry, Biophysics, Carbohydrate-binding Protein, Extracellular Matrix Protein, Glycobiology, Hyaluronate, Quartz Crystal Microbalance, Reflection Interference-Contrast Microscopy, Spectroscopic Ellipsometry, Supramolecular Structure

## Abstract

Mammalian oocytes are surrounded by a highly hydrated hyaluronan (HA)-rich extracellular matrix with embedded cumulus cells, forming the cumulus cell·oocyte complex (COC) matrix. The correct assembly, stability, and mechanical properties of this matrix, which are crucial for successful ovulation, transport of the COC to the oviduct, and its fertilization, depend on the interaction between HA and specific HA-organizing proteins. Although the proteins inter-α-inhibitor (IαI), pentraxin 3 (PTX3), and TNF-stimulated gene-6 (TSG-6) have been identified as being critical for COC matrix formation, its supramolecular organization and the molecular mechanism of COC matrix stabilization remain unknown. Here we used films of end-grafted HA as a model system to investigate the molecular interactions involved in the formation and stabilization of HA matrices containing TSG-6, IαI, and PTX3. We found that PTX3 binds neither to HA alone nor to HA films containing TSG-6. This long pentraxin also failed to bind to products of the interaction between IαI, TSG-6, and HA, among which are the covalent heavy chain (HC)·HA and HC·TSG-6 complexes, despite the fact that both IαI and TSG-6 are ligands of PTX3. Interestingly, prior encounter with IαI was required for effective incorporation of PTX3 into TSG-6-loaded HA films. Moreover, we demonstrated that this ternary protein mixture made of IαI, PTX3, and TSG-6 is sufficient to promote formation of a stable (*i.e.* cross-linked) yet highly hydrated HA matrix. We propose that this mechanism is essential for correct assembly of the COC matrix and may also have general implications in other inflammatory processes that are associated with HA cross-linking.

## Teaser

**Background:** The proteins pentraxin 3 (PTX3) and TNF-stimulated gene-6 (TSG-6) and the proteoglycan inter-α-inhibitor (IαI) are known to be involved in the stabilization of hyaluronan (HA)-rich extracellular matrices.

**Results:** PTX3 incorporation into HA matrices is tightly regulated.

**Conclusion:** PTX3, TSG-6, and IαI are sufficient to cross-link HA matrices.

**Significance:** The results provide mechanistic insights into the regulation of HA-protein interactions.

## Introduction

**FIGURE 1.**
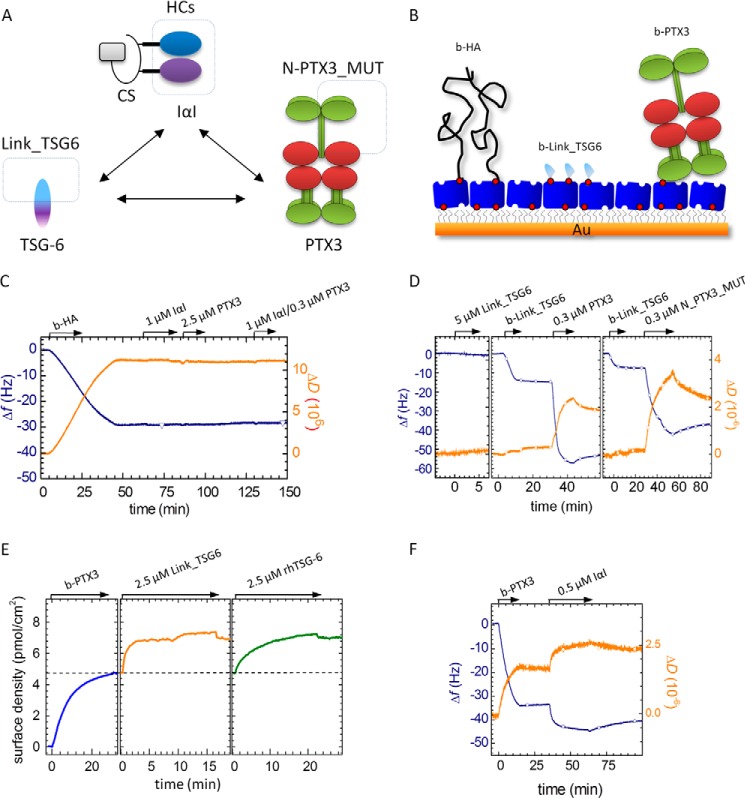
*A*, proteins involved in HA matrix stabilization. Ternary and quaternary structures are schematically shown, with the sizes of all proteins and their subunits approximately to scale and known interactions indicated by *arrows*. Subunits that were used separately in addition to the complete protein are enclosed within *dashed boxes. B*, schematic illustration of the platform for solid-phase binding assays. A gold support was modified with a protein-repellent oligoethylene glycol monolayer functionalized with biotin, followed by the formation of a dense monolayer of well oriented SAv. HA, Link_TSG6, or PTX3 was grafted to the SAv layer via biotin tags. HA chains were site-specifically functionalized with biotin at their reducing end and could therefore be immobilized at controlled orientation. Proteins were functionalized through primary amines and therefore might be immobilized in a variety of orientations. The thickness of the oligoethylene glycol monolayer and the dimensions of SAv, Link_TSG6, and the PTX3 octamer are drawn approximately to scale; the thickness of the HA brush and the mean distance between HA anchor points are reduced by 10–20-fold for illustrative purposes. *C*, interaction of PTX3, IαI, and a mixture of IαI/PTX3 with HA film. A control shows that 1 μm IαI, 0.3 μm PTX3, and a mixture of these proteins do not bind to HA in the absence of TSG-6. The start and duration of the incubation with different samples are indicated (*arrows*). After each incubation step, the solution phase was replaced by buffer. QCM-D did not show any significant interaction between the HA film (58 kDa) and IαI, PTX3, or a PTX3/IαI mixture. The employed HA films are easily permeated by the proteins (*cf.* Fig. 4*B*). The control measurements therefore also confirm that our streptavidin-coated surfaces are resistant to nonspecific binding of IαI or PTX3, alone or in a mixture. *D*, interaction of surface-bound Link_TSG6 with octamer-forming wild type PTX3 and dimer-forming N_PTX3_MUT. Interactions were measured by QCM-D. Biotinylated Link_TSG6 (b-Link_TSG6) but not Link_TSG6 without biotin was immobilized to a streptavidin monolayer, and binding of PTX3 constructs was monitored. The bulk PTX3 concentration refers to the molar concentration of PTX3 monomers. *E*, interaction of surface-bound PTX3 with Link_TSG6 and rhTSG-6. Interactions were measured by SE. b-PTX3 was immobilized, and binding of Link_TSG6 and rhTSG-6 was monitored. *F*, interaction of surface-bound PTX3 with IαI. Interactions were measured by QCM-D. b-PTX3 was immobilized, and binding of IαI was monitored. The *curves* shown in *C–F* are representative of sets of measurements performed at least in duplicate.

Many mammalian cells endow themselves with a pericellular matrix, also named the cellular coat or glycocalyx, which is located at the interface between the extracellular matrix and the cellular membrane and typically defined by its direct anchorage to the plasma membrane (1). Pericellular matrices have been reported for a variety of cells *in vivo* and *in vitro*, including fibroblasts (2, 3), chondrocytes (1, 4), epithelial cells (5), cancer cells (6), monocytes (7, 8), and endothelial cells (9, 10). The mechanisms of pericellular matrix cross-linking as well as how cross-linking alters the physico-chemical properties of the matrix and its functional activity remain poorly understood. An illustrative example of a cross-linked hyaluronan (HA)^5^ matrix is the cumulus cell·oocyte complex (COC) matrix. This extended visco-elastic matrix grows around the oocyte and cumulus cells a few hours before ovulation. The COC matrix remains around the oocyte not only in the course of ovulation but also during transport to the oviduct and is required for successful fertilization *in vivo* (11, 12). COC expansion is supported by the synthesis of HA (13, 14), and it has been suggested that HA in the matrix is organized in a hydrated meshwork via the cross-linking of the HA chains (15). Until now, three proteins have been described as essential for correct assembly of the COC matrix: inter-α-inhibitor (IαI (16, 17)), the secreted product of tumor necrosis factor-stimulated gene-6 (TSG-6 (18)), and pentraxin 3 (PTX3 (12, 19)). Currently, we have a limited understanding of how HA and these proteins interact with one another to stabilize the COC matrix. Fig. 1*A* outlines the ternary and quaternary structure of the three proteins.

TSG-6 is a multifunctional protein expressed under inflammatory conditions (15, 20) and by cumulus cells in the preovulatory follicle (20–25). It has numerous ligands, among which are HA (26, 27), IαI (21, 28, 29), and PTX3 (12, 30). TSG-6 is composed of two domains: the Link module with its HA-binding domain (31–34) and the CUB module (Protein Data Bank entry 2WNO) (35). In some cases, the Link module has been found to elicit biological responses similar to full-length TSG-6 (36–39). However, this is not the case for the interaction with HA; the Link module alone binds to HA in a simple manner, whereas the full-length protein binds to HA cooperatively, due to HA-induced TSG-6 oligomerization, a process that leads to cross-linking of HA (40).

IαI is a complex proteoglycan constitutively present in serum; it consists of two heavy chain subunits (HC1 and HC2) covalently linked via a chondroitin sulfate glycosaminoglycan to a light chain, bikunin (41–44). The interaction between IαI, TSG-6, and HA is known to result in the formation of covalent complexes (HA·HCs, TSG-6·HC) (21, 42, 43) and numerous non-covalent complexes. In the presence of IαI, the HA-binding and the cross-linking properties of TSG-6 are impaired (45). Instead, TSG-6 acts as a catalyst for the covalent transfer of HCs from the chondroitin sulfate chain of IαI onto HA (21). In the COC matrix, TSG-6 was found in two distinct populations: as a TSG-6·HC complex and in its native state (24, 25, 46). The covalent modification of the HA matrix with HCs is crucial for COC matrix assembly; genetic deficiency of either bikunin (which prevents assembly of intact IαI) or TSG-6 results in matrix instability and female infertility (17, 18, 47). However, the functional interplay between TSG-6 and IαI is not sufficient to stabilize the COC matrix because mice deficient in PTX3 are also unable to form stable cumulus matrix and are severely subfertile (48).

The soluble pattern recognition receptor PTX3, also called TSG-14, is a member of the pentraxin family (49, 50). The protein assembles into a multimeric complex of eight identical subunits stabilized by disulfide bonds (51, 52). Each protomer comprises a C-terminal pentraxin domain, sharing homology with the classical short pentraxins C-reactive protein and serum amyloid P component and a unique N-terminal region. PTX3 is an extracellular matrix protein that is expressed by a number of both somatic and immune cell types, including cumulus cells, in response to primary inflammatory stimuli and Toll-like receptor agonists (53–56). PTX3 fulfills important functions in fertility, vascular biology, and innate immunity (50, 57, 58). Its diverse functionality can be related to its complex structure. The PTX3 octamer has an elongated and asymmetric shape (52). It is composed of two differently sized globular lobes connected by a short stalk; the N- and C-terminal regions of PTX3 mediate binding to multiple ligands. Cumulus cells from *Ptx3*^−/−^ mice are unable to organize into a functional matrix, although the covalent modification of HA with HCs (HA·HC) remains unperturbed (12). The exogenous addition of PTX3 rescues COC matrix formation *ex vivo* (12, 30). Although PTX3 does not interact with HA, it has been suggested that its incorporation can be mediated by TSG-6 via its Link module domain through two distinct binding sites for HA and PTX3 (12). Coordinated expression of PTX3 and TSG-6 has been described in leukocytes and endothelial cells under inflammatory conditions (59) as well as in ovulation (12), suggesting that these proteins may co-localize in certain tissues (60) and cooperate *in vivo*.

More recently, in co-immunoprecipitation experiments on matrix extracts from murine COCs, PTX3 was shown to associate with HCs but not with the bikunin chain of IαI (48). Solid phase binding assays indicated that there is a direct interaction between the N-terminal domain of PTX3 and HCs. Moreover, recombinant constructs of this domain recapitulate the functional activity of PTX3 in ovulation (30, 48).

The oligomeric state of PTX3 is known to be functionally important; mutants of both the intact protein and its N-terminal region that form dimers were unable to rescue matrix assembly in *Ptx3*^−/−^ COCs, whereas mutants that form tetramers support formation of stable HA matrices (30, 51). PTX3 has been suggested to act as a HA cross-linker, which stabilizes the COC matrix (12, 21, 48). It was proposed that direct interaction of PTX3 with TSG-6 or HCs (or both) is critical for proper COC matrix assembly (48).

Despite the numerous data described above, a complete mechanistic picture of COC matrix formation and stabilization remains elusive. It has been our long term goal to be able to reconstitute the main features of this matrix *in vitro* based on a minimal set of molecular building blocks (*i.e.* to thereby demonstrate an understanding of this process). Here, we use films of surface-grafted HA and a range of surface-sensitive techniques to study in a well defined *in vitro* setting how IαI, PTX3, and TSG-6 affect the composition and cohesion of HA-rich matrices. We provide new insights into how these proteins act in a cooperative and coordinated fashion. We show that TSG-6 on its own cannot mediate PTX3 incorporation into HA films; nor can PTX3 be incorporated into HA films that have been previously exposed to IαI and TSG-6. Our data indicate that prior encounter of PTX3 and IαI is an essential requirement for successful incorporation of PTX3. The final quaternary complex assembled from HA, PTX3, TSG-6, and IαI (or parts of it, such as the HCs) can cross-link HA in a way that is different from the cross-linking that occurs with TSG-6 alone, such that the HA films remain strongly hydrated.

## EXPERIMENTAL PROCEDURES

### 

#### 

##### Protein and Hyaluronan Preparations

Wild type human TSG-6 Link module (Link_TSG6, 10.9 kDa) was expressed in *Escherichia coli* as described previously (61, 62). Biotinylated Link_TSG6 (b-Link_TSG6; species A) was made as before (63). Full-length recombinant human TSG-6 (rhTSG-6, 30.1 kDa) was expressed in *Drosophila* Schneider 2 cells and purified as described previously (64). IαI was purified from human serum as described (65) and confirmed to contain bikunin, HC1, and HC2 chains by Edman degradation and by liquid chromatography with tandem mass spectrometry (LC/MS/MS) of the major band cut out from an SDS-polyacrylamide gel (representing >95% of protein in the preparation). We assign a molecular mass value of 180 kDa for IαI (28) for molarity calculations, which is in good agreement with a size of ∼169 kDa determined by size exclusion chromatography with multiangle laser light scattering.^6^ Recombinant human PTX3 8-mer (PTX; 344 ± 7 kDa) was purified from a Chinese hamster ovary 3.5 cell line as described previously (57). A dimer-forming mutant of the PTX3 N-terminal domain (*i.e.* residues 18–170 of the preprotein; N_PTX3_MUT) was obtained by replacing cysteines at positions 47, 49, and 103 with serines as reported (39). Biotinylated PTX3 was obtained by modification of the recombinant protein with EZ-Link NHS-PEG4-Biotin (Thermo Scientific) according to the manufacturer's instructions; on average, 2 mol of biotin/mol of PTX3 protomer were incorporated, as quantified with the HABA-biotin quantitation kit (Thermo Scientific) used according to the manufacturer's instructions. Rat anti-human PTX3 monoclonal antibody (MNB4) was made as described previously (51, 66). Stock solutions of all proteins were aliquoted and stored at −20 °C. Protein solutions were thawed shortly before use and diluted to the desired concentrations.

Lyophilized HA, biotinylated at its reducing end and with well defined molecular masses of 1083 ± 53 kDa or 837 ± 54 kDa (*i.e.* two different batches of Select-HA B1000), was purchased from Hyalose (Oklahoma City, OK). For reconstitution, HA was taken up in ultrapure water at a stock concentration of 1 mg/ml, gently shaken overnight, aliquoted, and stored at −20 °C. Films of end-grafted HA on protein-repellent surfaces were assembled as described previously (28, 40, 45). For the solution-phase heavy chain transfer assays, HA decasaccharide (Hyalose) was biotinylated at the reducing end (b-HA_10_) by oxime ligation.^7^

A HEPES buffer (150 mm NaCl, 10 mm HEPES, pH 7.4, 2 mm CaCl_2,_ 5 mm MgCl_2_, in ultrapure water) was used throughout all measurements. Protein and HA solutions at their final concentrations were prepared in this buffer.

##### Quartz Crystal Microbalance with Dissipation Monitoring

QCM-D measurements were performed as described in detail elsewhere (67, 68). The QCM-D response is sensitive to the mass (including coupled water) and the viscoelastic properties of the surface adlayer. Measurements were performed with a Q-Sense E4 system (Biolin Scientific, Västra Frölunda, Sweden) in flow mode (69) with flow speeds of typically 20 μl/min and at a working temperature of 23 °C. QCM-D data were collected at six overtones (*n* = 3, 5, 7, 9, 11, and 13, corresponding to resonance frequencies of ∼15, 25, 35, 45, 55, and 65 MHz). Changes in dissipation and normalized frequency, Δ*f* = Δ*f_n_*/*n*, of the fifth overtone (*n* = 5) are presented unless otherwise stated. Adsorption and interfacial processes on gold-coated QCM-D sensors (QSX301, Biolin Scientific) were monitored *in situ* with subsecond time resolution (70).

For sufficiently rigid monolayers of proteins, the film thickness can be estimated to within an error of typically <20%, from *d* = −*C*/ρ × Δ*f*, where ρ = 1.0 g/cm^3^ is the density of the bulk solution and *C* = 18.06 ng/cm^2^/Hz is the mass sensitivity constant for a sensor with a fundamental resonance frequency of 4.95 MHz (71). For submonolayers of globular proteins, this calculus provides an effective thickness that is smaller than the extension of the protein perpendicular to the surface.

##### Spectroscopic Ellipsometry (SE)

SE measures changes in the polarization of light upon reflection at a planar surface. We employed SE (M2000V; Woollam, Lincoln, NE) *in situ* to quantify adsorbed biomolecular masses in a time-resolved manner, as described in detail elsewhere (40, 45, 69). Gold-coated silicon wafers were used as substrates and installed in a custom-built open cuvette with continuously stirred sample solution (∼150 μl).

##### Solution-phase Heavy Chain Transfer Assays

Heavy chain transfer assays with catalytic amounts of TSG-6 were carried out as described by Rugg *et al.* (28). Briefly, 1.8 μm IαI was incubated with 0.27 μm TSG-6 with excess b-HA_10_ (20 μm) in the presence or absence of 1.8 μm PTX3 at 37 °C in HEPES-buffered saline with 5 mm MgCl_2_. Reactions were halted at 0, 1, 2, and 4 h by the addition of SDS-sample loading buffer and boiling for 3 min. Samples were run on a 10% Tris-Tricine SDS-polyacrylamide gel and transferred to a nitrocellulose membrane. In contrast to the previously reported assay (28), we used HA oligosaccharides in biotinylated form as a substrate for HC transfer. This modification enabled highly specific detection of the b-HA_10_·HC product (and thus monitoring of HC transfer) by fluorescently labeled streptavidin. Specifically, fluorescence of streptavidin-conjugated Alexa 488 (Invitrogen) was visualized using a ChemiDoc Imager (Bio-Rad), and band intensities were quantified using ImageJ software.

##### Western Blotting

Samples of ∼150-μl volume were extracted from the ellipsometry cuvette and stored frozen in aliquots of 30 μl until required. The collected material was analyzed for the presence of TSG-6, IαI and the subunits of IαI by Western blot (samples were electrophoresed on 4–12% NuPAGE BisTris gels with SeeBlue Plus2 prestained standard (Invitrogen)), using RAH-1 and anti-IαI (DAKO) antibodies, respectively, with a LI-COR Odyssey system as described previously (45). The presence of PTX3 in the eluates was assessed by Western blot with chemiluminescence detection. Briefly, proteins in the eluates were recovered using 15 μl of StrataClean resin (Agilent Technologies) and incubated for 15 min at room temperature under agitation, followed by a water wash. The resin-bound material was denatured and reduced by heating at 70 °C for 10 min in sample loading buffer containing DTT (Invitrogen). Proteins were separated by SDS-PAGE on 10% BisTris gels (Invitrogen), using MOPS SDS running buffer, and transferred onto 0.45-μm PVDF membranes. PTX3 was detected with the MNB4 rat monoclonal antibody (500 ng/ml), followed by a secondary anti-rat IgG HRP conjugate (1:5000 dilution; GE Healthcare). Chemiluminescence was recorded on a Chemidoc system (Bio-Rad), following the addition of enhanced chemiluminescence substrate (Millipore).

##### Colloidal Probe Reflection Interference-Contrast Microscopy

The thickness of surface-bound HA films (837 kDa) was determined by triple-wavelength colloidal probe RICM, as described previously (67, 72). Naked polystyrene microspheres of ∼25-μm diameter (Polysciences, Eppelheim, Germany) or SAv-functionalized polystyrene microspheres of ∼22.7-μm diameter (Spherotech, Lake Forest, IL) were used as colloidal probes. HA films were assembled on gold-coated glass coverslips, using custom-built open cells with an internal volume of 50 μl. Protein solutions at desired concentrations were added, and the incubation time was set to 5 h. Colloidal probes were added shortly before acquisition of RICM images.

##### Functionalization of Colloidal Beads with HA Films

Streptavidin-functionalized polystyrene microspheres were used. The stock solution at 0.5% (w/v) concentration was washed by three cycles of adding a 5-fold volume excess of ultrapure water, centrifugation at 5500 × g for 10 min, and removal of the supernatant. End-biotinylated HA (837 kDa) was added to 0.5% (w/v) of particles at a final concentration of 50 μg/ml (this would correspond to a bead surface area of 1 nm^2^/HA chain if all available chains were grafted to beads) in HEPES buffer and incubated under agitation for 30 min at room temperature. Unbound HA was eluted by five cycles of washing. The HA-functionalized beads were used immediately after preparation.

##### Quantification of the Mobility of Colloidal Probes by RICM

The motion of microspheres was monitored by RICM imaging at a wavelength of 490 nm, at a rate of 8 frames/s and an exposure time of 100 ms, for 62.5 s. From the images, variations in the bead position were quantified in two perpendicular directions (*x*, *y*) parallel to the surface, with a resolution of the order of 1 nm through custom-made image analysis software. From the evolution of the spatial position as a function of time *t*, the mean square displacement (MSD) was calculated. In some assays, the microspheres were found to undergo a directed lateral motion, in addition to random stochastic motion, presumably driven by convection or by gravity on a slightly tilted sample, or by lateral drifts of the sample stage. To correct for the directed motion, we subtracted a linear fit from the *x*(*t*) and *y*(*t*) data sets before computing the MSD. The statistical uncertainty of the MSDs increases with lag time τ. We considered only MSDs up to τ = 0.625 s for further analysis. Based on the number of data points per trace (500 frames), we estimate a statistical uncertainty of ±18% for the MSDs at this lag time.

## RESULTS

We employed purpose-designed solid-phase binding assays to assess molecular and supramolecular interactions. Gold-coated surfaces were functionalized with an oligoethylene glycol coating exposing biotin groups and a dense monolayer of streptavidin (SAv), as described previously (40) (Fig. 1*B*). The SAv layer displays sites for the stable immobilization of biotinylated biomolecules and allows oriented immobilization when the biomolecules are site-specifically labeled (*e.g.* for HA). The underlying oligoethylene glycol ensures that only biotinylated species can bind directly to the surface (*i.e.* the binding is specific). QCM-D and SE were used to monitor the binding events. Here, SE provided quantification of the adsorbed biomolecular mass per unit surface area. The QCM-D response is sensitive to the amount of adsorbed ligand (including coupled solvent), with a negative frequency shift Δ*f* typically correlating with a mass increase, and sensitive to mechanical properties as well as morphological features of the biomolecular film, typically reflected in the dissipation shift Δ*D* (71).

### 

#### 

##### PTX3 Interacts with Link_TSG6, rhTSG-6, and IαI in the Absence of HA

PTX3 does not interact directly with HA (Fig. 1*C*). The incorporation of the protein into HA-rich matrices (in particular into the COC matrix) must hence be mediated by other molecular players that interact with HA. TSG-6 (12) and the HCs of IαI (48) have been proposed to be involved in this process.

To confirm the functionality of our protein samples, we probed the interaction of PTX3 with TSG-6 in the absence of HA. The binding of wild type PTX3, which forms an octamer (52) of approximately cuboid shape with a size of 9.6 × 14.3 × 26 nm^3^, and a mutant of its N-terminal domain whose oligomerization is restricted to dimers (N_PTX3_MUT) to biotinylated Link_TSG6 (b-Link_TSG6) was first tested by QCM-D (Fig. 1*D*). Clear responses in frequency (Δ*f*) and dissipation (Δ*D*) upon incubation of the SAv-coated surface with b-Link_TSG6 (Fig. 1*D*, *middle* and *right*) confirmed binding of the protein. No desorption was observed upon rinsing in buffer, and Link_TSG6 lacking biotin did not bind (Fig. 1*D*, *left*), confirming that b-Link_TSG6 was firmly and specifically immobilized through biotin. The addition of wild type PTX3 (Fig. 1*D*, *middle*) or N_PTX3_MUT (Fig. 1*D*, *right*) resulted in rapid and pronounced binding. The interaction between Link_TSG6 and PTX3 was specific (*i.e.* PTX3 did not bind to a Link_TSG6-free surface; Fig. 1*C*) and rather stable (*i.e.* only a minor fraction of the proteins desorbed within 10 min after rinsing in buffer). N_PTX3_MUT bound with similar stability as the intact wild type protein, consistent with the observation that it is the N-terminal domain of PTX3 that mediates binding to TSG-6 (30, 60).

The dissipation shift for the protein films was sufficiently small, such that effective film thicknesses can be estimated from the frequency shifts (71) (*i.e.* as an additional element to assess the quality of the proteins and their interaction). More specifically, the interactions in this binding assay should generate (sub)monolayers of b-Link_TSG6 and the associated PTX3 constructs. For partial (or complete) monolayers of globular proteins, the effective thickness should be smaller than (or comparable with) the extension of the protein perpendicular to the surface. The frequency shifts at the end of the incubation processes correspond to effective film thicknesses of 2.5 and 1.4 nm for b-Link_TSG6 (Fig. 1*D*, *middle* and *right*, respectively; the difference may reflect batch-to-batch variations in the biotinylation of b-Link_TSG6 leading to different surface coverages), 10 nm for PTX3/b-Link_TSG6 (Fig. 1*D*, *middle*), and 8 nm for N_PTX3_MUT/b-Link_TSG6 (Fig. 1*D*, *right*). These values are indeed smaller than or comparable with the protein dimensions (32, 40, 52) consistent with the formation of (sub)monolayers.

A reverse approach was used in a complementary SE assay, such that biotinylated PTX3 (b-PTX3) was first immobilized at a monomer surface density of 4.8 pmol/cm^2^, corresponding to 1 PTX3 octamer/280 nm^2^ (Fig. 1*E*). Here, binding of both Link_TSG6 and rhTSG-6 (which is not available in a biotinylated form) could be tested. In contrast to QCM-D, SE can provide quantitative information about surface coverage and binding stoichiometry. The addition of both TSG-6 constructs at identical molar bulk concentrations yielded comparable molar surface densities corresponding to ∼50% of the PTX3 monomer surface density. Assuming that the TSG-6 constructs bind as monomers to the individual binding sites on PTX3 and based on our previous observations (30, 58), this suggests that each PTX3 octamer has four TSG-6 binding sites. However, we cannot exclude the possibility that some binding sites are inaccessible due to steric constraints imposed by the surface and the dense arrangement of proteins. Most of the bound protein could not be eluted by rinsing in buffer, indicating that binding is of high affinity, consistent with the QCM-D data (Fig. 1*D*). Although Link_TSG6 and rhTSG-6 bound in similar amounts and with similar stability, they differ in their adsorption kinetics; Link_TSG6 bound rapidly with an initial binding rate that suggests mass transport-limited binding, whereas rhTSG-6 bound much more slowly. The distinctly different binding kinetics might well reflect differences in the mode of binding of these TSG-6 constructs, such as previously reported for their binding to HA (40). However, we cannot fully exclude the possibility that the decreased binding rate for rhTSG-6 is due to steric effects; the solid support and crowding might partially mask TSG-6 binding sites in the PTX3 monolayer, rendering binding of rhTSG-6 (∼30 kDa) more difficult than that of the smaller Link_TSG6 (∼11 kDa). Taken together, these data provide clear evidence for a strong interaction between TSG-6 and PTX3. The results are consistent with a binding mediated through the Link module of TSG-6 to a dimer of the N-terminal region of PTX3, as has been concluded previously (30, 60).

An additional QCM-D assay with surfaces exposing a monolayer of b-PTX3 (Fig. 1*F*) confirmed that IαI binds to PTX3, as expected (30). As observed for TSG-6, binding was rather stable, with only a minor fraction of the proteins being desorbed within 10 min after rinsing in buffer.

##### Ternary Interaction between TSG-6 Constructs, PTX3, and HA

**FIGURE 2.**
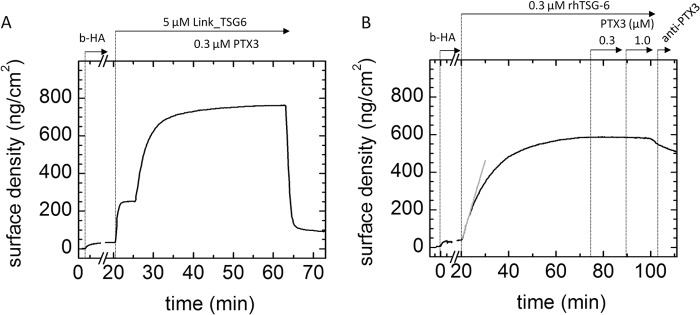
**PTX3 does not incorporate into HA films via full-length rhTSG-6, but it does via Link_TSG6.** The HA film (837 kDa) was first loaded with Link_TSG6 (*A*) and rhTSG-6 (*B*). After equilibrium was established, free TSG-6 protein remained in solution. The addition of PTX3 revealed fast adsorption to the Link_TSG6-loaded HA film but not to the rhTSG-6-loaded HA film. The *gray solid line* in *B* is a linear fit revealing an initial binding rate of 44 ng/cm^2^/min. The addition of 0.08 μm anti-PTX3 antibody (MNB4) in the rinsing phase in *B* did not affect the unbinding curve. The SE *curves* shown are representative of sets of measurements performed at least in duplicate.

The ternary interaction between HA, PTX3, and TSG-6 could lead to incorporation of PTX3 into HA matrices only if the HA and PTX3 binding sites on TSG-6 do not interfere with each other. To test whether this is the case, we performed a sequential binding assay (Fig. 2). First, the HA film was incubated with Link_TSG6 at a bulk concentration of 5 μm (Fig. 2*A*). The surface density of adsorbed material at equilibrium was 215 ± 16 ng/cm^2^, corresponding to an occupancy of 1 Link_TSG6/1 HA decasaccharide, in good agreement with previous work (40). An excess of Link_TSG6 was maintained in the bulk solution, and no rinsing step was included, to prevent desorption of Link_TSG6, which would otherwise occur rapidly (40). The addition of PTX3 at a bulk monomer concentration of 0.3 μm resulted in a large increase in the adsorbed mass. The surface density of incorporated material was 418 ± 55 ng/cm^2^. Because PTX3 alone does not interact with HA (Fig. 1*C*), we conclude that its incorporation is Link_TSG6-mediated, as proposed before (12, 21). Hence, the binding sites for HA and PTX3 on Link_TSG6 do not overlap, allowing Link_TSG6 to act as a linker for PTX3 incorporation into HA matrices.

**FIGURE 3.**
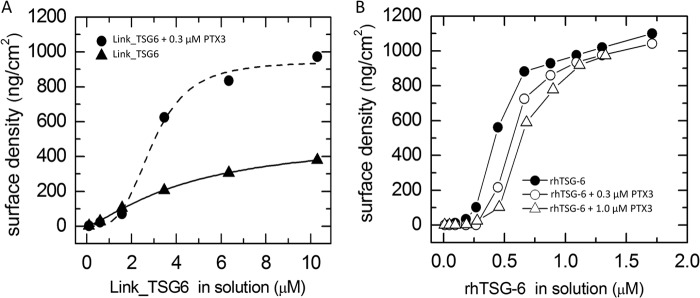
**PTX3 differentially modulates the interactions of Link_TSG6 and full length TSG-6 with HA.** *A*, PTX3 modulates Link_TSG6 interaction with HA. Binding isotherms obtained from titration of Link_TSG-6 into an HA film, monitored by SE, in the presence or absence of 0.3 μm PTX3 in the solution phase. A fit (*solid line*) of the titration data for Link_TSG6 (*filled triangles*) with the Hill equation gave a Hill exponent close to 1.0, confirming a simple non-cooperative interaction between HA and Link_TSG6. In the presence of PTX3, the interaction is more complex. The sigmoidal shape of the data (*filled circles*) indicates cooperative binding. A fit with the Hill equation (*dashed line*) indeed provides a Hill exponent of 3.6 (*i.e.* much larger than 1.0). *B*, competition of PTX3 and HA for TSG-6 binding. Binding isotherms obtained from titration of rhTSG-6 into an HA film, alone (*filled circles*) and in the presence of 0.3 μm (*filled triangles*) or 1 μm (*empty triangles*) PTX3 in the solution phase. At a bulk rhTSG-6 concentration of 0.45 μm, an ∼3-fold decrease in the surface density of adsorbed rhTSG-6 can be detected in the presence of 0.3 μm PTX3. At 1 μm PTX3, the decrease in binding becomes even more pronounced.

Surprisingly, PTX3 did not show any measurable binding if the HA film was preloaded with full-length TSG-6 instead of Link_TSG6. Consistent with this, subsequent incubation with the MNB4 antibody, which recognizes the N-terminal domain of PTX3, did not lead to any increase in binding signal (*i.e.* it did not appreciably affect the rhTSG-6 unbinding curve) (Fig. 2*B*). However, from Fig. 1, *D* and *E*, as well as previous reports (12, 30, 60), we know that rhTSG6 can interact with PTX3 in the absence of HA. Thus, it would appear that the interaction of rhTSG-6 with PTX3 is perturbed by HA. To shed light on this, we performed binding assays in which the HA film was exposed to a constant amount of PTX3, along with increasing concentrations of either Link_TSG6 (Fig. 3*A*) or rhTSG-6 (Fig. 3*B*). For comparison, both TSG-6 constructs were also titrated into the HA film in the absence of PTX3 and were found to exhibit distinct binding (Fig. 3), as has been noted previously (40); binding of rhTSG-6 to HA is characterized by a pronounced positive cooperativity, and the *K*_0.5_ is about 5-fold lower than for Link_TSG6.

However, the amount of adsorbed Link_TSG6 was greater in the presence of PTX3 (Fig. 3*A*), consistent with the first binding assay (Fig. 2*A*). It is notable that enhanced binding was observed when Link_TSG6 was present in solution at >2 μm but not with lower Link_TSG6 concentrations. Moreover, the binding isotherm in the presence of PTX3 (Fig. 3*A*, *filled circles*) exhibited a pronounced sigmoidal shape, where a fit with the Hill equation gave an exponent of 3.6, indicative of cooperativity, with *K*_0.5_ = 2.9 μm and Γ_max_ = 945 ng/cm^2^. In contrast, a fit to the data from Link_TSG6 alone gave an exponent of 1.3 (*i.e.* close to 1.0) and *K*_0.5_ = 4.6 μm, which is in agreement with previous reports (40) and is indicative of simple non-cooperative binding. Thus, the cooperative binding behavior in the presence of PTX3 must somehow originate from the interaction of Link_TSG6 with PTX3. More specifically, binding of Link_TSG6 to PTX3 or, alternatively, of the Link_TSG6·PTX3 complex to HA must be cooperative.

Removal of proteins from the bulk solution after the titration assays (not shown) revealed distinct rates of protein dissociation from the HA film. The dissociation rate of Link_TSG6 alone (31 ± 2 × 10^−3^ s^−1^; not shown) was comparable with previously reported rates on polymeric HA and oligo-HA films (40). The dissociation rate in the presence of PTX3 (14.7 ± 0.6 × 10^−3^ s^−1^) was 2 times lower than for Link_TSG6 alone. The decrease in desorption rate might indicate that the PTX3·Link_TSG6 complex binds to the films in a multivalent manner or that PTX3 enhances the HA binding activity of Link_TSG6.

Within the experimentally accessible range of rhTSG-6 concentrations (up to 2 μm), PTX3 inhibited protein binding to HA in a dose-dependent manner. The addition of a monoclonal anti-PTX3 antibody (MNB4) to the protein-loaded HA films at the end of the titrations did not result in any significant response (not shown), confirming that the bound protein material consisted exclusively of rhTSG-6. Apparently, PTX3 competes with HA for the binding of rhTSG-6. As a consequence, we conclude that PTX3 cannot be incorporated into HA matrices through rhTSG-6 alone, contrary to what has been proposed in the literature (12) based on the observation that Link_TSG6 can bind to PTX3 and HA.

##### Interaction of PTX3 with HA Films That Were Pre-exposed to TSG-6 and IαI

**FIGURE 4.**
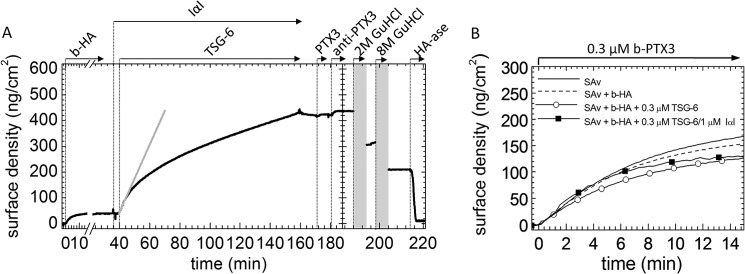
**PTX3 does not bind to HA films that had previously been exposed to a mixture of IαI/TSG-6.** *A*, binding assays by SE. 1 μm IαI and 0.3 μm TSG-6 were sequentially added to the HA film without premixing. In this case, the film contains an additional fraction of non-covalently but stably bound protein (45). The *gray solid line* is a linear fit revealing an initial binding rate of 13 ng/cm^2^/min. Incubation with 0.3 μm PTX3 does not affect the surface density of the film. The lack of a significant response upon incubation with 0.08 μm anti-PTX3 antibody (MNB4) confirms the absence of PTX3 binding. The *curve* shown is representative of a set of measurements performed in duplicate. *B*, HA films are permeable to PTX3. b-PTX3 was added to SAv-covered surfaces without any further functionalization (*solid line*) or in the presence of HA (837 kDa) films with a surface density of 35 ± 5 ng/cm^2^. HA films were presented pure (*dashed line*) or following exposure to 0.3 μm rhTSG-6 (*solid line* with *open circles*) or to a mixture of 1 μm IαI and 0.3 μm TSG-6 (premixed for 1 min before the addition to the HA film; *solid line* with *filled squares*). Only initial binding is shown. Binding with similar rates is observed for all surfaces. Because PTX3 alone did not show binding on any of these surfaces, the binding of b-PTX3 must occur via the biotin moiety to SAv, indicating that all HA films are permeable to PTX3.

PTX3 is also known to bind to intact IαI (30, 48), and it has been proposed that PTX3 incorporates into HA matrices through interaction with HCs (48). The latter can be covalently transferred from IαI to HA via a reaction that is catalyzed by TSG-6 (21). To test this hypothesis, we first exposed HA films to a mixture of TSG-6 and IαI and then studied PTX3 binding (Fig. 4*A*).

Incubation with 0.3 μm PTX3 and subsequently with anti-PTX3 antibody (MNB4) revealed that the TSG-6/IαI-exposed film did not bind significant amounts of PTX3. This unexpected finding seems to indicate that when IαI forms ternary interactions with TSG-6 and HA, it is no longer able to bind PTX3. Alternatively, one may argue that PTX3 binding is simply limited by the access of the protein to the interior of the HA film. In this regard, PTX3 forms octamers in solution (52) that are significantly larger than TSG-6 or IαI.

Thus, to test whether PTX3 is sterically excluded from HA films, we performed permeation assays using b-PTX3 (Fig. 4*B*). The assay exploits the fact that only a small fraction (∼1%) of the biotin binding sites on the SAv monolayer that accommodates the HA film are occupied by b-HA (67). Biotinylated PTX3 that diffuses through the HA film should hence find plenty of sites to bind through its biotin moieties. b-PTX3 readily bound to a plain SAv monolayer and to SAv monolayers covered with HA films with a surface density of 35 ± 5 ng/cm^2^ that were pure or had been incubated with 0.3 μm rhTSG-6, alone or in a mixture with 1 μm IαI. Importantly, the initial b-PTX3 binding rates for all of the HA films tested here did not differ significantly from the initial rate on the plain SAv surface (Fig. 4*B*), indicating that the different HA films do not significantly affect the accessibility of b-PTX3 to SAv. Clearly, all of these HA films retained good permeability to PTX3. Taken together, we must conclude, based on the data presented in Figs. 1*C*, 2*B*, 3*B*, and 4, that neither pure HA films nor HA films treated with TSG-6 or with a mixture of TSG-6 and IαI present appropriate PTX3 binding sites.

##### How to Incorporate PTX3 into HA Films?

**FIGURE 5.**
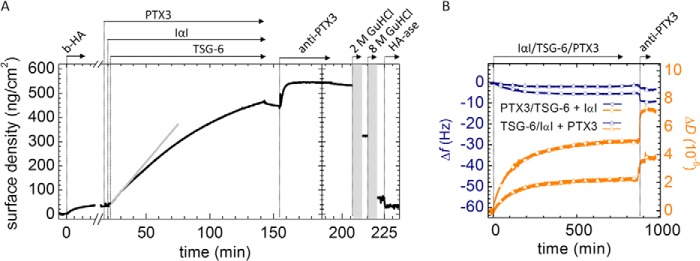
**PTX3 incorporates into HA films when presented in a ternary mixture with TSG-6 and IαI.** *A*, binding assay by SE. HA films were first exposed to 0.3 μm PTX3. After 2 min of incubation, 1 μm IαI was added, and after another 2.5 min, 0.3 μm rhTSG-6 was added. Binding ensued after the addition of rhTSG-6; the *gray solid line* is a linear fit revealing an initial binding rate of 6 ng/cm^2^/min. The protein mixtures were incubated with the HA films for 2 h. Subsequent binding of anti-PTX3 antibody, incubated at 0.08 μm, indicates successful incorporation of PTX3. The *curve* shown is representative of a set of measurements performed in duplicate. *B*, binding assay by QCM-D. Δ*f* (*blue lines*) and Δ*D* (*orange lines*) are shown. Mixtures of PTX3, rhTSG-6, and IαI were exposed to HA films at final protein concentrations of 1.0, 0.6, and 0.2 μm, respectively. In one case (*circles*), PTX3 was first mixed with TSG-6 for 2 h and then with IαI for another 1 h (all at room temperature) before exposure to HA. In the other case (*triangles*), TSG-6 and IαI were mixed first (for 2 h), and then PTX3 was added (for 1 h) before exposure to HA. Clear QCM-D responses upon the subsequent addition of anti-PTX3 antibody, incubated at 0.08 μm, indicated successful incorporation of PTX3.

The absence of PTX3 binding in the above-described assays disproves existing hypotheses about the mechanism by which PTX3 is incorporated into HA matrices (12, 48). On the other hand, PTX3 has been shown to be an essential component in the formation of the COC matrix (12). So how is PTX3 incorporated into HA films? We hypothesized that PTX3 must encounter IαI and/or TSG-6 prior to interaction with HA in order for PTX3 to be incorporated into HA assemblies. To test this hypothesis, we first added PTX3 and IαI at bulk concentrations of 0.3 and 1 μm, respectively, to an HA film. As anticipated, this mixture did not show any HA binding activity (Fig. 1*C*) (45). Second, TSG-6 was added at a bulk concentration of 0.3 μm. This protein did start a binding reaction (Fig. 5*A*). After 2 h of incubation, all proteins in the soluble phase were removed. Following this, the anti-PTX3 antibody (MNB4) did bind to the film. In contrast, the same antibody did not bind to an HA film that contained TSG-6 (Fig. 2*B*). This provides strong evidence that co-incubation of PTX3 in a ternary mixture with IαI and TSG-6 promotes PTX3 incorporation into the HA film.

We carried out additional measurements (by QCM-D; Fig. 5*B*) to test whether the sequence of protein encounter is critical for PTX3 incorporation. In one case, PTX3 was first mixed with TSG-6 and then with IαI before the mixture was exposed to the HA film. In a second measurement, TSG-6 and IαI were mixed first, and then PTX3 was added before exposure to HA. In both cases, PTX3 was incorporated into the HA films, as evidenced by subsequent binding of anti-PTX3 antibody. We conclude that the order of protein encounter is not critical for PTX3 incorporation.

It is difficult to quantify the amount of incorporated PTX3 based on bound antibody; accessibility to PTX3 in the HA film might be limited, and it is also not clear if the antibody can bind simultaneously to all monomers in the PTX3 octamer. However, by assuming a stoichiometry of one antibody per PTX3 monomer and using a molecular mass of the antibody of 150 kDa, a lower limit for the amount of incorporated PTX3 in Fig. 5*A* can be estimated as 0.6 pmol/cm^2^. For comparison, the density of TSG-6 when incubated alone at the same concentration would be about 20-fold higher.

Our data do not provide a full picture of the exact composition of the polysaccharide-protein assembly that forms upon interaction of the ternary protein mixture with HA. Considering that the four starting molecules can engage in a variety of homotypic and heterotypic interactions, it is also difficult to suggest which molecule/molecular complex acts as the ligand for PTX3. However, we can extract some information about the functional role of PTX3 by analyzing the kinetics of the binding reaction and the stability of binding from the ternary protein mixture.

The initial rate of the binding reaction in the presence of all three proteins (Fig. 5*A*, *straight gray line*) was more than 5 times smaller than the initial binding rates of TSG-6 alone (Fig. 2*B*, *straight gray line*) and about 2 times smaller than the reaction rate for the binary mixture of TSG-6 and IαI (Fig. 4*A*, *straight gray line*). This indicates that the propensity of IαI to (partially) impair the binding of TSG-6 to HA (45) is retained and perhaps even enhanced in the presence of PTX3.

**FIGURE 6.**
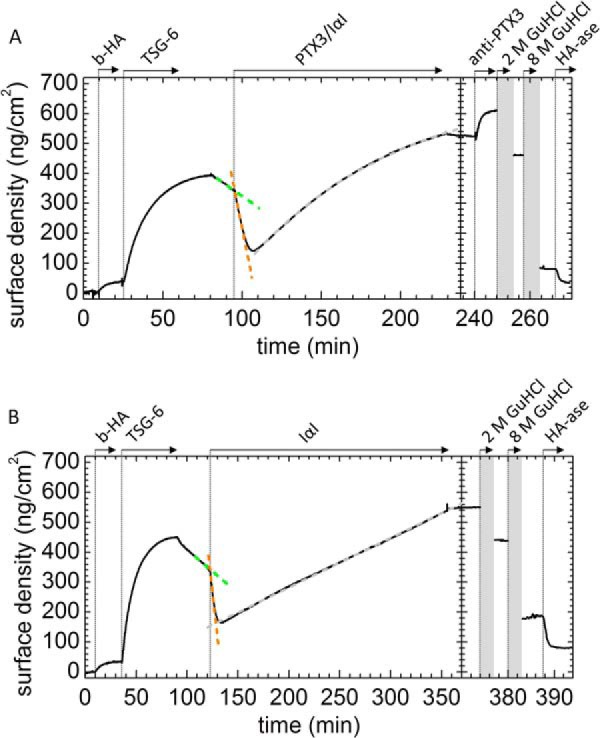
**PTX3 in a mixture with IαI incorporates into TSG-6-loaded HA films.** *A*, the HA film was first loaded with 0.3 μm TSG-6, excess protein was removed from the solution phase, and a mixture of 0.3 μm PTX3, 1 μm IαI was added (premixed for 1 min). The addition of the PTX3/IαI mixture first enhanced desorption until a mass fraction of 35% remained, and thereafter incorporation of material into the HA film started. The addition of anti-PTX3, after the removal of excess protein in the solution phase, confirmed PTX3 incorporation into the film. Only a small fraction of about 12% could be eluted with 2 m GdnHCl, whereas most material was eluted with 8 m GdnHCl. A fraction of 10% remained bound in 8 m GdnHCl but could be largely digested by hyaluronidase. The increase in the desorption rate upon the addition of IαI/PTX3 can be appreciated from the linear fits to the data shortly before (*green dashed line*; 3.7 ng/cm^2^/min) and after (*red dashed line*; 27 ng/cm^2^/min) protein addition. The adsorption process setting in 10 min after incubation with IαI/PTX3 was fitted by an exponential (*gray dashed line*). The fit revealed a maximal surface density of 660 ng/cm^2^ and a half-time of about 90 min. The *curve* shown is representative of a set of measurements performed in duplicate. *B*, equivalent measurement with IαI instead of a mixture of IαI and PTX3, with linear fits to the data shortly before (green *dashed line*; 2.9 ng/cm^2^/min) and after (orange *dashed line*; 31 ng/cm^2^/min) IαI addition, reproduced from Fig. 1*B* in Ref. 45. In this case, the adsorption process, commencing 10 min after incubation with IαI, could be fitted with a straight line (*gray dashed line*).

To test whether PTX3 influences the competition between IαI and HA for TSG-6, we performed another sequential incubation assay (Fig. 6*A*); here IαI and PTX3 were premixed and then exposed to a TSG-6-loaded HA film (where the TSG-6 had been removed from the bulk solution and the HA-associated protein was beginning to dissociate). Linear fits allowed us to approximate the unbinding rates just before (3.7 ng/cm^2^/min; *green dashed line*) and after the addition of IαI/PTX3 (27 ng/cm^2^/min; *red dashed line*). Comparison with the displacement induced by IαI alone (Fig. 6*B*; reproduced from Ref. 45) did not reveal any significant difference. Also, the fraction of stably but non-covalently bound material that persisted after incubation with IαI and PTX3 was comparable with the fraction displaced by IαI alone (30 and 35%, respectively). Hence, PTX3 does not affect the propensity of IαI to displace TSG-6 from HA. More generally, this finding would indicate that PTX3 does not influence the initial interaction between TSG-6 and IαI.

Approximately 10 min after the start of incubation with IαI and PTX3 (Fig. 6*A*, at 107 min), an increase in the surface density was observed. We had already seen a similar effect with IαI alone (Fig. 6*B*) (45) and concluded that this response relates to transfer of HCs onto HA, accompanied by the incorporation of several non-covalently bound protein species into the HA film. The overall similarity in the rates and magnitudes of binding suggests that similar processes occur also in the presence of PTX3, yet a detailed comparison of the binding curves reveals distinct shapes; the curve for IαI alone (Fig. 6*B*) (45) was linear over more than 3 h of incubation, whereas the binding curve for the mixture of IαI and PTX3 (Fig. 6*A*) is well approximated by an exponential with a half-time of 90 min. Previously, we have proposed that the linear response is a signature of the TSG-6-mediated transfer of HCs from IαI onto HA (45), with TSG-6 acting as a catalyst (23). In this context, the exponentially decaying binding rate in Fig. 6*A* might indicate that, in the presence of PTX3, TSG-6 is consumed in the HC transfer reaction. In other words, we propose that PTX3 inhibits recycling of TSG-6.

**FIGURE 7.**
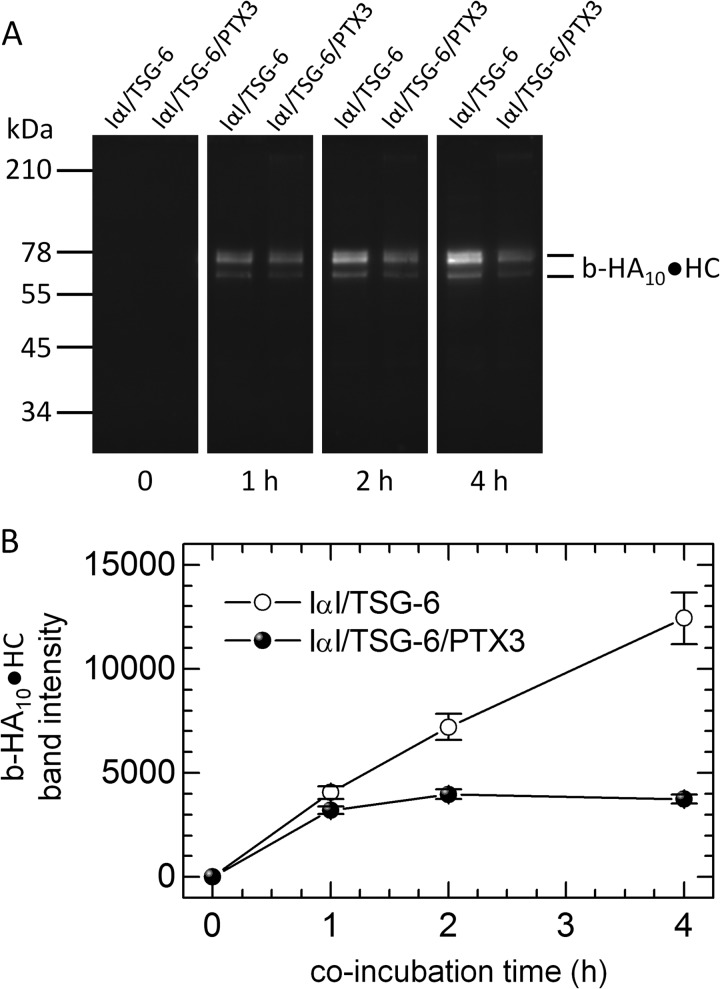
**PTX3 inhibits the catalytic activity of TSG-6 in HC transfer.** In a solution-phase assay, 0.27 μm TSG-6, 1.8 μm IαI, and 20 μm b-HA_10_ were co-incubated with or without 1.8 μm PTX3 for various times and subsequently analyzed by Western blots with streptavidin-conjugated Alexa 488, which recognizes biotin in b-HA_10_·HC complexes. *A*, Western blot with co-incubation times indicated. *B*, densitometric analysis of Western blots. *Error bars*, S.E. from three blots. Data are representative of two independent experiments.

To test this hypothesis, we performed complementary HC transfer assays in solution, in which catalytic amounts of TSG-6 (28) were co-incubated with IαI and a biotinylated HA oligosaccharide (b-HA_10_) in the absence/presence of PTX3 (Fig. 7). In the absence of PTX3, transfer rates remained virtually unchanged throughout the total 4-h incubation time (based on the visualization of b-HA_10_·HC species; see Fig. 7*A*), consistent with the linear response in Fig. 6*B*. In contrast, in the presence of PTX3, transfer proceeded throughout the first hour but then essentially halted. These data provide independent confirmation that PTX3 reduces the ability of TSG-6 to transfer heavy chains and are fully consistent with our proposal that PTX3 inhibits recycling of TSG-6. It should be noted that the experiments in Figs. 6 and 7 were conducted at different temperatures (24 and 37 °C, respectively) and that the rate of encounter between HA and the proteins is higher with HA in the solution phase, which probably explains the different time scales over which this inhibition was observed.

##### Stability of PTX3 Incorporation and Effect of PTX3 on HA Film Composition

**FIGURE 8.**
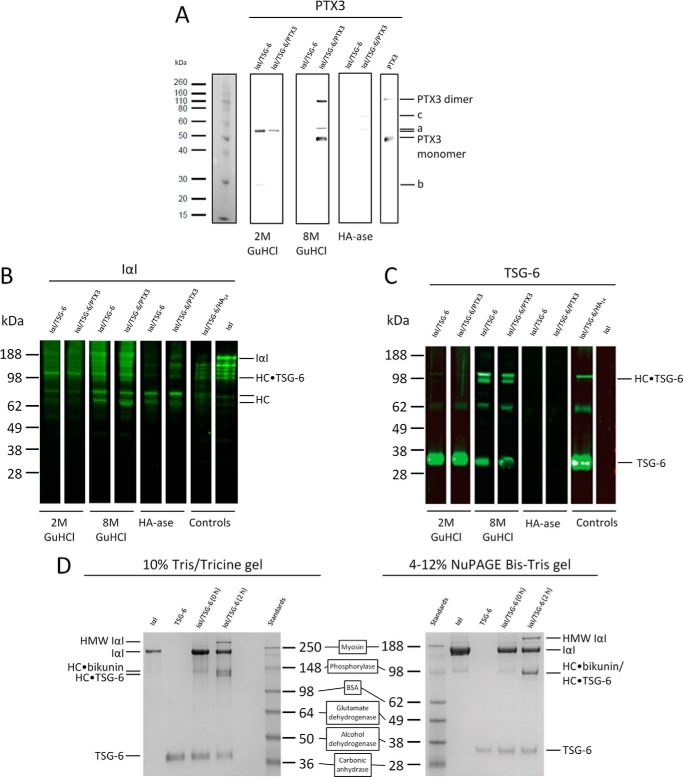
**Western blot analysis of protein material incorporated into HA films.** *A–C*, HA films were incubated with proteins in binary (IαI/TSG-6) and ternary (IαI/TSG-6/PTX3) mixtures, as shown in Figs. 4*A* and 5, respectively. Western blots were made from fractions obtained by stepwise elution with 2 and 8 m GdnHCl and by digestion with hyaluronidase (*HA-ase*). Collected material was analyzed by Western blots with anti-PTX3 (*A*), anti-IαI (*B*), and anti-TSG-6 (*C*) antibodies. The control reaction mix of TSG-6, HA_14_, and IαI is expected to contain a total amount of 100 ng of TSG-6 and 25 ng of IαI, the control lane for PTX3 is expected to contain 500 pg of PTX3, and the detection limits are estimated to be around 5 ng for TSG-6, 0.5 ng for IαI, and 50 pg for PTX3. *D*, direct comparison of IαI and TSG-6 proteins running in 4–12% NuPAGE BisTris gels (used in *B* and *C*) and 10% Tris/Tricine gels (used by Rugg *et al.* (28)), stained with Coomassie Blue. The same standards were used for both gel types (as indicated), and their apparent molecular masses were assigned following the manufacturer's indications for NuPAGE BisTris 4–12 with MES (as in *B* and *C*) and Tris-glycine gels (as in Ref. 28), respectively. IαI and TSG-6 were mixed (1.8 and 2.7 μm, respectively) and co-incubated at 4 °C using the standard conditions (as described in Ref. 28). Immediately after mixing (0 h), bands for intact IαI and TSG-6 are dominant; after 2 h of co-incubation, additional bands for HC·bikunin/HC·TSG-6 appear. In the 10% Tris/Tricine gel, IαI, HC·bikunin, the HC·TSG-6 doublet, and TSG-6 run at apparent molecular masses of ∼220, ∼130, ∼120, and ∼38 kDa, respectively, consistent with Rugg *et al.* (28), where these had been identified by Edman degradation and mass spectrometry. In the 4–12% NuPAGE BisTris gels, the apparent molecular masses for IαI and TSG-6 are ∼170 and ∼34 kDa, respectively; the HC·bikunin and HC·TSG-6 species run together with an apparent molecular mass of ∼98 kDa. *HMW I*α*I*, a high molecular weight form of IαI with three or four HCs attached, which is a minor species within the IαI preparation purified from serum that also forms as a by-product of HC·TSG-6 complex formation (see Ref. 29).

To analyze the composition of the HA film after protein incubation, we performed Western blot analyses (with anti-PTX3, anti-TSG-6, and anti-IαI antibodies) of material collected from the incubation assays in Figs. 4*A* and 5 after exposure sequentially to 2 m GdnHCl, 8 m GdnHCl, and hyaluronidase. The anti-PTX3 antibody MNB4 (Fig. 8*A*) revealed the two strongest bands to be in the 8 m GdnHCl eluate from the ternary protein mixture; the apparent molecular masses of these bands (about 45 and 90 kDa) were identical to that of the PTX3 monomer and dimer that are present in a control sample. No bands were found at these positions in the 2 m GdnHCl eluates and the hyaluronidase digests (or the 8 m GdnHCl eluate from the IαI/TSG-6 binary mixture). This indicates that most, if not all, PTX3 is very tightly yet non-covalently bound to the HA matrix. Some immunoreactive bands were observed at 55 and 28 kDa in samples from 2 m GdnHCl washes (Fig. 8*A*, labeled as *a* and *b*, respectively). These might correspond to the heavy and light chains, respectively, of antibody leftovers in the measurement chamber from the injections performed in Figs. 4*A* and 5. Also a band at about 65 kDa (Fig. 8*A*, labeled as *c*) is present, probably due to sample contamination with BSA, that is nonspecifically recognized by the applied antibody.

The staining patterns with the anti-TSG-6 and anti-IαI antibodies (Fig. 8, *B* and *C*) were consistent with our previous reports (45) (*i.e.* rhTSG-6 protein and HC·TSG-6 were found in all GdnHCl eluates but not in the hyaluronidase digest, whereas HC was also found in the hyaluronidase digest). Notably, the blots of eluates and digests retrieved from the PTX3-containing HA films were very similar to the corresponding ones from the PTX3-free HA film. Apparently, the presence of PTX3 does not appreciably affect the incorporation of TSG-6, the HCs of IαI, or HC·TSG-6 complexes into the HA film.

In the Western blots in Fig. 8, *B* and *C*, we assign the bands at apparent molecular masses of ∼170 and ∼34 kDa to IαI and TSG-6, respectively; these bands were clearly the dominant ones in control gels (using the same gel type and molecular weight standards) with Coomassie Blue staining of the IαI and TSG-6 preparations (Fig. 8*D*, *right*), demonstrating the purity of the two protein preparations. However, when using molecular masses from Ref. 28 for the SeeBlue Plus2 standards in the context of a 10% Tris/Tricine gel, an apparent molecular mass of ∼220 kDa is observed for the same IαI preparation (Fig. 8*D*, *left*); similar values have been reported previously by us and others (28, 73–75). Thus, it can be seen that the gel system used and the masses assigned to standards have a large influence on the apparent molecular weight determined for IαI from SDS-PAGE; as noted above, the mass of IαI determined in solution by a biophysical method is ∼169 kDa (see “Experimental Procedures”).

Additional bands, located between 100 and 160 kDa, that are hardly visible with Coomassie Blue staining are rather pronounced within the IαI control in the Western blot developed with the anti-IαI antibody (Fig. 8*B*). It seems likely that these species represent minor traces of IαI degradation and that the enhanced relative intensity in the Western blot originates from an overproportional sensitivity of the polyclonal anti-IαI antibody for the degraded species. Other faint bands are also detectable slightly above the IαI band on Coomassie Blue staining (Fig. 8*D*, *right*), which we suggest represent IαI-like proteins with three or four heavy chains attached to the bikunin-chondroitin sulfate proteoglycan; we have referred to these previously as high molecular weight IαI, which are low abundance forms of IαI found in plasma (42) and which are also generated as by-products during HC·TSG-6 complex formation (see Fig. 8*D*) (29, 42, 45).

##### Structural Role of PTX3

PTX3 has been suggested to act as an HA cross-linker that stabilizes the COC matrix (12, 48). Furthermore, the oligomeric state of PTX3 is known to be functionally important: mutants of both PTX3 and its N-terminal region that form dimers were unable to rescue matrix assembly in *Ptx3*^−/−^ COCs; in contrast, the wild type octamer-forming protein and tetramer-forming mutant constructs, made in the context of both full-length PTX3 and its N-terminal domain, did support formation of stable HA matrices (30).

**FIGURE 9.**
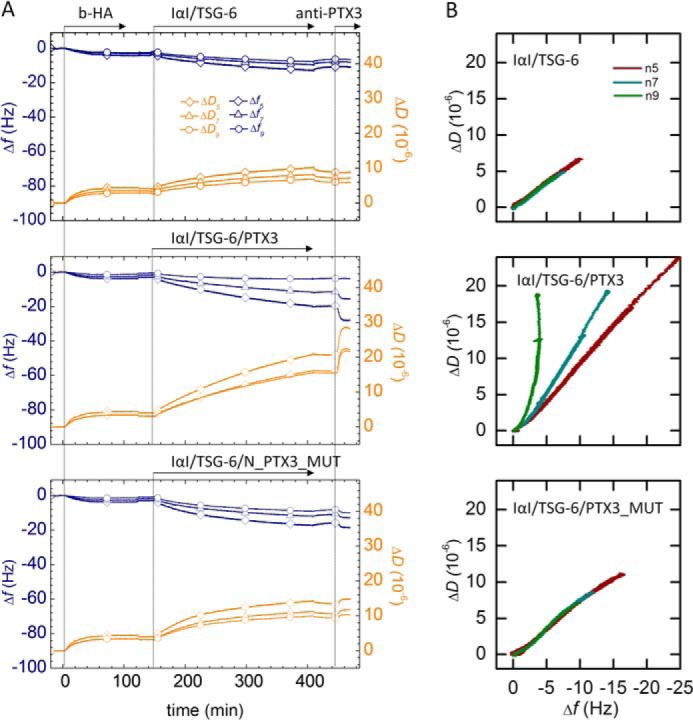
**Effect of PTX3 on HA film morphology.** *A*, binding assays by QCM-D. Δ*f* and Δ*D* at three selected overtones (*n* = 5, 7, and 9) are shown. HA films were first exposed to the protein mixtures 1.0 μm IαI, 0.3 μm TSG-6; 0.3 μm PTX3, 1.0 μm IαI, 0.3 μm TSG-6; or 0.3 μm N_PTX3_MUT, 1.0 μm IαI, 0.3 μm TSG-6, with all proteins premixed (in the indicated order) shortly before the addition to the HA films. After removal of excess protein from bulk solution, the incorporation of PTX3 into the HA matrices was confirmed by the addition of 0.1 μm anti-PTX3 (MNB4). Films exposed to the binary IαI/TSG-6 mixture did not show any significant response to anti-PTX3. A decrease in Δ*f* (*blue curves*) accompanied by an increase in Δ*D* (*orange curves*) for both ternary mixtures evidences anti-PTX3 binding, confirming that both PTX3 and N_PTX3_MUT can be incorporated into HA films. *B*, parametric plots reveal morphological differences. Plots of Δ*D versus* Δ*f* are shown, for the incubation of HA films with binary and ternary protein mixtures. Data were taken from *A*, and Δ*D* and Δ*f* were offset to zero shortly before the start of protein incubation. This parametric plot provides a qualitative fingerprint of how the mechanical properties of the HA matrix evolve upon protein incorporation. For the binary mixture and the ternary mixture containing N_PTX3_MUT, all overtones produce a roughly linear response. The slope is comparable between overtones and also between the two protein mixtures. The latter indicates that N_PTX3_MUT, although incorporated into the film, does not affect the film morphology drastically as compared with the binary protein mixture. The response for the ternary protein mixture containing wild type PTX3 is distinct, with a large spread between the three overtones and non-linear shapes for *n* = 7 and 9. This indicates that incorporation of PTX3 does affect the film morphology appreciably.

To test whether PTX3 affects the morphology of HA meshworks, we monitored the response of HA films to incorporation of different protein mixtures by QCM-D (Fig. 9*A*). The response to a binary mixture of IαI and TSG-6 was compared with that to ternary protein mixtures including either PTX3 or N_PTX3_MUT (150–410 min). The signals observed upon the addition of the anti-PTX3 antibody (at 445 min) provide evidence that not only wild type PTX3 but also N_PTX3_MUT bind.

Parametric plots of Δ*D versus* Δ*f* are useful to detect changes in the morphology or mechanical properties of surface-confined films (76–78). The strong similarities in such plots (Fig. 9*B*) for films containing IαI and TSG-6 and films containing additionally N_PTX3_MUT and the clear differences compared with films containing intact PTX3 provide strong evidence that PTX3, but not its dimeric N-terminal region, modifies the morphology and/or mechanical properties of the HA film as compared with the binary protein mixture of IαI and TSG-6. We stress that the qualitative analysis of Δ*D versus* Δ*f* plots is appropriate for films of arbitrary morphological or mechanical complexity. It is also robust because it makes use of the raw data alone. In contrast, we did not attempt to obtain quantitative insight into the nature of the changes occurring in the presence of intact PTX3 from QCM-D data; because these HA films are generally very soft (as can be appreciated from the large magnitude of the dissipation shifts or, more accurately, the large Δ*D*/−Δ*f* ratio (71)), this would require multiparameter data fitting through viscoelastic modeling (*i.e.* a method that has more stringent requirements for the film properties (71), which we are not certain to be fulfilled for our films).

**FIGURE 10.**
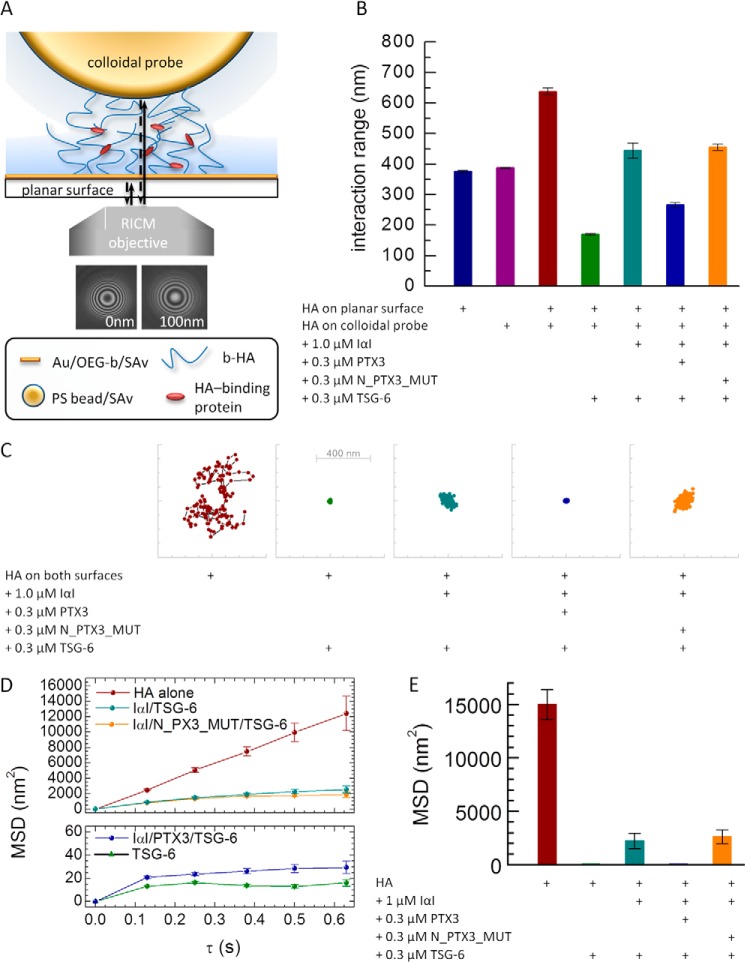
**Effect of proteins on HA film thickness and cross-linking.** *A*, sketch of the experimental setup. HA films (837 kDa) were formed separately on a planar surface and on colloidal probes, and the probes were then added to the planar surface. The probe-surface distance (interaction range) and the in-plane thermal motion of the probe were measured by RICM, for pure HA films and in the presence of protein mixtures. *B*, interaction range. Proteins were incubated for 2 h at the indicated concentrations. Proteins were added to the HA film in sequential order: first IαI, either alone or in a mixture with a PTX3 construct (octamer-forming wild type (PTX3) or dimer-forming mutant (N_PTX3_MUT); premixed for 1 min) and thereafter TSG-6. *Error bars*, S.E. of 10 measurements with different colloidal probes on the same sample; data are representative of at least two sets of experiments on different samples. *C*, representative traces of the thermally driven random in-plane movement of a bead's center over a period of 62.5 s for pure HA films on the planar surface and on the colloidal probe, alone and in the presence of protein mixtures. The scales in the *x* and *y* direction span a range of 800 nm. *D*, lateral MSD as a function of lag time τ extracted from the curves shown in *C. Error bars* correspond to statistical uncertainties for a given measurement. *E*, MSD for τ = 0.625 s. *Error bars*, S.E. of three measurements with different colloidal probes on the same sample.

Instead, we quantified the variations in HA film thickness upon protein incorporation by RICM. To this end, films with 837-kDa HA were prepared on SAv-functionalized glass coverslips as well as on SAv-coated polystyrene colloidal probes. The interaction between a planar HA film on the glass coverslip and the HA film on the colloidal probe (with the colloidal probe hovering on top of the glass surface due to weak gravitational forces) was then monitored by RICM (Fig. 10*A*) in the absence and in the presence of selected proteins. The distance between the bead and the glass coverslip, displayed in Fig. 10*B*, hence represents the interaction range across the two films.

The interaction range in the case of pure HA films was 640 ± 28 nm. Control measurements of the thickness of planar HA films on the glass coverslips, measured with uncoated polystyrene beads, and of curved HA films on the colloidal probe, measured with an uncoated glass coverslip, gave similar thicknesses, each of ∼380 nm. The measured interaction range for two HA films is hence about 1.7-fold larger than the thickness of the individual HA films. To a first approximation, the interaction range for the encounter of two identical HA films would be expected to be twice the thickness of individual HA films. However, considering that the two films are likely to exhibit some degree of interpenetration, the measured value of 1.7 appears reasonable (79).

In the presence of TSG-6, the interaction range decreased dramatically, from 640 ± 28 to 169 ± 10 nm. In the presence of IαI and TSG-6, the interaction range was 444 ± 67 nm, in agreement with our previous results (45), and for the ternary protein mixture, this number decreased to 266 ± 10 nm. Clearly, the ternary mixture of TSG-6/IαI/PTX3 produced a decrease in the interaction range that was less pronounced than for TSG-6 alone yet more pronounced than for the binary mixture of TSG-6/IαI. In contrast, when intact PTX3 in the ternary mixture was replaced by N_PTX3_MUT, the interaction range (456 ± 24 nm) was comparable with the binary IαI/TSG-6 mixture. This provided a first clear indication that the dimer-forming mutant of the N-terminal domain and the octameric wild type PTX3 impact the HA film in distinct ways.

The RICM assay with two opposed HA films can also provide insight into the interaction between HA films. To this end, we tracked the in-plane motion of HA-coated beads in the presence of selected proteins for typically 1 min. Fig. 10*C* reveals that the thermally driven random motion of the beads is sensitive to the presence of proteins. For example, for a pure HA film, the motion trace covered a surface area of 500 × 500 nm^2^, whereas it was confined to a 2500-fold smaller area in the presence of TSG-6 alone and in the presence of the ternary mixture of IαI/TSG-6/PTX3. For the binary IαI/TSG-6 mixture, the covered surface area was also reduced, albeit to a lesser extent (about 25-fold). Interestingly, N_PTX3_MUT did not show the same response as the intact PTX3 protein when at a monomer concentration equivalent to that of PTX3 octamer. Instead, the motion of the colloidal probe was comparable with that of the binary IαI/TSG-6 mixture, indicating that oligomers larger than dimers are required for the reduction of mobility.

For quantitative comparison, we also computed the MSD (Fig. 10, *D* and *E*) and analyzed the dependence of the MSD on lag time. For pure HA films, we found the MSD to depend linearly on lag time (Fig. 10*D*). A linear response is expected for free diffusive motion, indicating that the lateral motion of the HA-coated colloidal probe is not spatially constrained by the HA-coated glass coverslip, as expected for two mutually repelling films.

In stark contrast, the MSD was virtually constant for lag times above τ = 0.125 s in the presence of either TSG-6 alone or the ternary TSG-6/IαI/PTX3 mixture (*i.e.* the lateral motion of the beads was spatially confined to a surface area of about 70 × 70 nm^2^). Moreover, gentle agitation of the fluid phase with a pipette did not displace the bead (not shown). Such a response would only be expected if the interaction between the two HA films is attractive, and we propose that it arises from the protein-mediated cross-linking of HA. The strong effect of TSG-6, which we had previously established to be a potent cross-linker of HA (40), supports this hypothesis. Our data, therefore, provide indirect evidence that a ternary protein mixture containing PTX3 can also cross-link HA. It is interesting that although films containing PTX3 and those containing only TSG-6 are both stably cross-linked, they are condensed to very different degrees (Fig. 10*B*). The plateau in the MSD, observed in both scenarios (Fig. 10*D*), indicates that the ensemble of the protein-mediated interactions between (and within) the two opposing HA films is sufficiently strong to promote the formation of a hydrogel (*i.e.* an HA matrix where the intermolecular interactions are sufficiently strong to prevent flow).

Over the experimentally accessible range of lag times, the MSD for the binary mixture of IαI and TSG-6 increased continuously albeit sublinearly with lag time. This indicates that the beads' diffusive motion is not entirely free and is affected by attractive interactions between the HA films. The elevated magnitude of the MSD (as compared with TSG-6 alone and with the ternary TSG-6/IαI/PTX3 mixture; Fig. 10*E*) and the lack of a plateau in the MSD (Fig. 10*D*) indicate that the attractive interaction must be relatively weak in this case. The lateral distances explored by the bead over an observation time of 1 min were below 100 nm (Fig. 10*C*). This distance is more than an order of magnitude shorter than the contour length of the HA molecules. It would hence be possible that, even in this case, the confinement of the beads' motion is due to formation of a hydrogel, with the displacement of the bead being constrained by tethering (*i.e.* cross-linking) to a few HA molecules in the planar HA film. However, because no clear plateau in the MSD could be observed, the response would also be consistent with other subdiffusive motions that do not require a stable contact between the two HA films (*i.e.* where the bead could explore longer distances when given more time). Our data, therefore, do not provide conclusive evidence for the formation of a hydrogel by binary TSG-6/IαI mixtures.

Remarkably, the MSD trace for IαI/TSG-6/N_PTX3_MUT was comparable with that of the binary IαI/TSG-6 mixture (Fig. 10, *D* and *E*), indicating that PTX3 dimers, although present in the HA films (Fig. 9*A*), do not promote cross-linking. This confirms that strong PTX3-mediated cross-linking requires PTX3 oligomers larger than dimers, in good agreement with *ex vivo* data, where it was shown that tetrameric PTX3 is the minimal oligomeric size required for the stabilization of the COC matrix (30).

## DISCUSSION

We have investigated the interactions between well defined HA films and an ensemble of three proteins that are essential for the integrity of the COC matrix: IαI, PTX3, and TSG-6. By varying the sequence of protein addition to HA and by using a range of surface-sensitive techniques, we provide new insights into how these proteins integrate their actions in matrix assembly. We have shown that TSG-6 alone cannot mediate PTX3 incorporation into HA films; nor does PTX3 bind to HA films that contain the products of the ternary interaction between IαI, TSG-6, and HA, among which are the covalent HC·HA and HC·TSG-6 complexes. Our data indicate that prior encounter between IαI, alone or jointly with TSG-6, and PTX3 leads to successful PTX3 incorporation into HA matrices and demonstrate that native octameric PTX3 (but not dimeric N_PTX3_MUT) in cooperation with TSG-6 and IαI can create a cross-linked HA matrix.

### 

#### 

##### TSG-6-mediated Binding of PTX3 to HA

**FIGURE 11.**
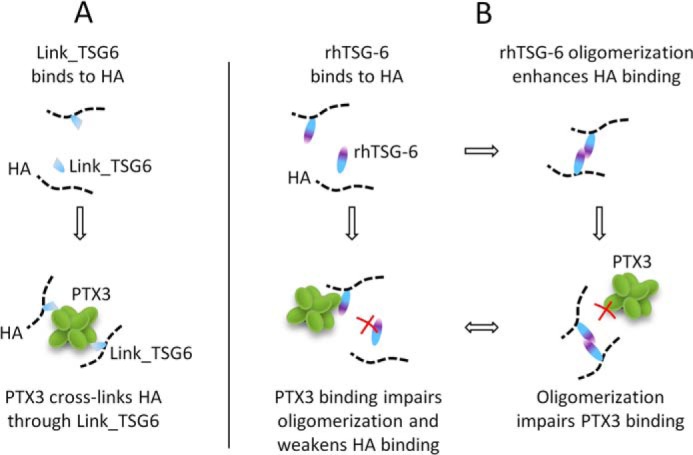
**Interaction of PTX3 with TSG-6 and HA.** Sketches illustrate proposed mechanisms for Link_TSG6 (*A*) and rhTSG-6 (*B*).

Salustri *et al.* (12) hypothesized that TSG-6 mediates the incorporation of PTX3 into HA matrices. More recently, surface plasmon resonance assays have demonstrated that the binding of TSG-6 to PTX3 is mediated by its Link module and that the binding sites for PTX3 and HA are different (60). Our data on incorporation of PTX3 into HA films via Link_TSG6 are consistent with these findings (Figs. 2*A* and 3*A*; illustrated in Fig. 11*A*).

However, although Leali *et al.* (60) reported similar affinities of Link_TSG6 and rhTSG-6 for PTX3 (0.3 and 0.6 μm, respectively) and although we confirmed that both TSG-6 constructs can bind PTX3 rather tightly (Fig. 1*E*), we could not find any evidence for the incorporation of PTX3 into HA films via full-length TSG-6 (Figs. 2*B* and 3*B*). Instead, PTX3 appeared to inhibit the binding of TSG-6 to HA at the concentrations tested here (Fig. 3*B*). What is the reason for this discrepancy? Our previous work provided evidence that HA induces oligomerization of rhTSG-6 but not Link_TSG6, and we proposed that the CUB_C domains of rhTSG-6 associate during oligomerization (40). Although the CUB_C domain does not seem to be directly involved in the interaction between TSG-6 and PTX3 (60), it is possible that HA-induced oligomerization of TSG-6 creates steric constraints that impair PTX3 binding. According to this model, the binding of PTX3 to full-length TSG-6 would probably compete directly with TSG-6 oligomerization. We also proposed that oligomerization is responsible for the enhanced binding of rhTSG-6 to HA, as compared with Link_TSG6 (40). Thus, PTX3 could indirectly weaken the interaction between TSG-6 and HA. The data presented in Fig. 3*B* would be consistent with such a scenario, illustrated in Fig. 11*B*.

We stress in particular that the Link_TSG6 concentrations required to promote efficient incorporation of PTX3 into HA films exceeded those experimentally accessible for our *in vitro* assays with rhTSG-6. They also far exceeded the maximum reported *in vivo* concentrations of TSG-6 (80). Therefore, we conclude that rhTSG-6 alone is unlikely to serve as an efficient linker for the binding of PTX3 to HA under physiologically relevant conditions.

##### The Display of PTX3 Binding Sites in HA Matrices Appears to Be Tightly Regulated

We also demonstrated that the incubation of HA films with a binary mixture of IαI and TSG-6 leads to the formation of covalent HA·HC complexes and to the stable, but non-covalent, incorporation of several other molecules and molecular complexes into the matrix; these include (presumably HC-bound) TSG-6 and covalent HC·TSG-6 complexes (40). Considering that PTX3 is known to engage in rather strong binary interactions with TSG-6 as well as with HCs (12, 30, 60), it is quite remarkable that such a matrix is inert to PTX3 binding (Fig. 4). Scarchilli *et al.* (48) suggested that TSG-6 might favor the interaction of PTX3 with HCs in HC·TSG-6 complexes. We indeed found HC·TSG-6 being incorporated into HA films (Fig. 8*B*), but apparently the intermolecular interactions and the local arrangement of the molecules and their complexes in the matrix are organized in such a way that all PTX3 binding sites are unavailable.

PTX3 was incorporated into HA films only if it was premixed with TSG-6 and IαI prior to the addition to HA (Fig. 5) or if PTX3 and IαI were premixed and then added to a TSG-6-containing HA matrix (Fig. 6*A*). Clearly, protein encounters, and perhaps the encounter of PTX3 with IαI in particular, play a critical role in PTX3 incorporation. Moreover, our observation that the TSG-6 displacement rate in sequential incubation assays did not depend on PTX3 (Fig. 6) suggests that the initial interaction between IαI and TSG-6 is not influenced by PTX3.

The presence of PTX3 did not have a detectable impact on the qualitative composition of species detected in HA films (Fig. 8, *B* and *C*). This result is consistent with *in vivo* studies where HC transfer was found to be unaffected in *Ptx3*^−/−^ mice (12). The comparison of the putative HC transfer kinetics in the sequential incubation assays (Fig. 6) and in the solution-phase transfer assays (Fig. 7) indicated that TSG-6 is not recycled as an HC transfer enzyme in the presence of PTX3. A plausible explanation for the inhibition of the enzymatic function of TSG-6 could be that TSG-6 is not released from the matrix upon transfer of HCs but remains in a PTX3-bound state. More specifically, we propose that the formation of HC·TSG-6 complexes and subsequent transfer of HCs to HA takes place in the context of a complex of PTX3 and IαI with TSG-6. The reorganization of this complex upon transfer of HCs from the C4S chain of IαI to TSG-6 and subsequently to HA leads to the integration of PTX3 into the HA matrix.

Although the specific interactions through which PTX3 integrates into the HA matrix remain elusive, it is clear from our data that the sequence of encounters between proteins and HA is the determinant for the correct assembly of a matrix that contains PTX3, IαI, TSG-6, and HA. A remote analogy between the formation of HA-based extracellular matrices and intracellular signaling pathways can perhaps be drawn; both processes exhibit spatio-temporal regulation and involve a hierarchy of interactions (81). We have already proposed the concept of a hierarchy of interactions for the assembly of HA matrices in our previous work (45) and hypothesized that HC·HA complexes act as central players in matrix assembly. Here, it appears that PTX3 has an elevated status in the sense that its incorporation into the matrix is only realized under very specific conditions.

##### Structural Role of PTX3 in the Assembly of COC Matrix

We previously provided evidence that TSG-6 alone can cross-link, and hence stabilize, HA matrices (40). Why then are the expression of PTX3 and the presence of IαI essential for the assembly of a functional COC matrix? Here, we demonstrate that different combinations of proteins result in HA films with distinct morphologies (Fig. 10). This finding may have important implications for our understanding of the formation and molecular organization of the COC matrix.

HA films with incorporated PTX3 became less collapsed/condensed than HA films containing just TSG-6 (Fig. 10*B*). Upon incubation with the IαI/TSG-6/PTX3 mixture, the HA film thickness decreased to 260 nm, whereas TSG-6 alone led to a thickness of 170 nm. Considering the amount of HA and proteins in the films, estimated to be 450 ng/cm^2^ (Fig. 5*A*) and 600 ng/cm^2^ (Fig. 2*B*), we can calculate a water content of 98.3% and 96.5%, respectively. Clearly, both films remain highly hydrated and hence permeable even to large proteins (Fig. 4*B*).

Direct measurements of the interaction forces between an HA-coated bead and an HA-coated planar film (*e.g.* by atomic force microscopy or optical tweezers) should in the future provide direct and quantitative information about the cross-linking strength. The presence of weak cross-links that are reversible on the time scale of interest is likely to have a major relevance to the process of COC expansion; opening of transient cross-links can support integration of newly synthesized HA chains into the matrix, thereby facilitating matrix growth. Given the large size of individual HA chains, a rather low concentration of cross-linkers would be required to stabilize the assembly.

To summarize, we propose that weak, transient cross-linking by products of the quaternary HA/IαI/TSG-6/PTX3 interactions can simultaneously support two processes, matrix stabilization and matrix expansion. Furthermore, orchestration of the encounter between the different protein species in time and space may lead to spatio-temporal modulation of HA matrix morphology. These processes may be of primary relevance not only in formation of the COC matrix but also in inflammatory processes that are accompanied by the coordinated expression of TSG-6 and PTX3 and/or encounter with IαI (82).

## Footnotes

6 D. C. Briggs, J. J. Enghild, and A. J. Day, unpublished data.

7 D. Thakar, E. Migliorini, L. Coche-Guerente, R. Sadir, H. Lortat-Jacob, D. Boturyn, O. Renaudet, P.Labbé, and R. P. Richter, submitted for publication.

## Footnotes

5 The abbreviations used are:

HA: hyaluronan
b-HA_10_: HA decasaccharide (Hyalose) biotinylated at the reducing end
HC: heavy chain
IαI: inter-α-inhibitor
COC: cumulus cell·oocyte complex
Link_TSG6: HA binding domain of TSG-6 protein
TSG-6: secreted product of tumor necrosis factor-stimulated gene-6
rhTSG-6: recombinant human TSG-6
SAv: streptavidin
GdnHCl: guanidine hydrochloride
RICM: reflection interference-contrast microscopy
PTX3: pentraxin 3
b-PTX3: biotinylated PTX3
QCM-D: quartz crystal microbalance with dissipation monitoring
SE: spectroscopic ellipsometry
Tricine: *N*-[2-hydroxy-1,1-bis(hydroxymethyl)ethyl]glycine
BisTris: 2-[bis(2-hydroxyethyl)amino]-2-(hydroxymethyl)propane-1,3-diol
MSD: mean square displacement.
